# AI-enabled cardiovascular devices: a lifecycle playbook for evidence, change control, and post-market assurance

**DOI:** 10.3389/fdgth.2026.1785381

**Published:** 2026-03-19

**Authors:** Nurittin Ardic, Rasit Dinc

**Affiliations:** 1 Med-International UK Health Agency Ltd., Leicestershire, United Kingdom; 2 INVAMED Medical Innovation Institute, New York, NY, United States

**Keywords:** artificial intelligence, cardiovascular devices, change control, clinical validation, governance, post-market surveillance, software as a medical device

## Abstract

AI-enabled cardiovascular devices are increasingly used in imaging, physiological signal analysis, and clinical decision support systems. Despite growing clinical adoption, requirements for evidence generation, software change management, and post-deployment assurance remain fragmented across jurisdictions and are often difficult to translate into operational processes within healthcare organizations. This review synthesizes common foundations of software as a medical device (SaMD) oversight and compares key regulatory expectations regarding cardiovascular AI across the United States, the European Union, and the United Kingdom, with emphasis on the entire clinical lifecycle from pre-deployment assessment to post-market monitoring. Across jurisdictions, convergent operational requirements emerge: First, external validation reflecting real-world heterogeneity with assessment beyond discrimination, including calibration and clinically relevant threshold performance. Second, structured governance of software updates with predefined limits and verification/validation activities. Third, transparency and traceability documentation supporting safe use, accountability, and auditability. Finally, continuous post-market surveillance with longitudinal performance monitoring across clinically relevant subgroups. These requirements are translated into a set of practical implementation artifacts, including a transparency document template, a site acceptance testing protocol, a governance workbook aligned with predefined change planning concepts, a monitoring dashboard specification linking key performance indicators to predefined actions, and an accountability framework outlining organizational responsibilities. Representative cardiovascular use cases (CT-based functional assessment, ECG-based screening and triage, and echocardiographic quantification) illustrate how modality-specific sources of variability impact monitoring priorities and governance considerations. This synthesis supports procurement, governance, and quality assurance activities for AI-enabled cardiovascular devices while maintaining alignment with contemporary methodological standards and regulatory expectations.

## 1 Introduction

AI-enabled cardiovascular devices are increasingly used in imaging, physiological signal analysis, and clinical decision support. While many machine learning systems exhibit strong discrimination during development and internal validation, performance often varies after deployment in heterogeneous clinical settings. Differences in patient populations, disease prevalence, acquisition protocols and hardware, labeling practices, and workflow integration can lead to dataset drift between development and real-world use, resulting in downstream impacts on model calibration, error profiles, and clinical utility (1–3). In cardiovascular care, these challenges are amplified by modality-specific heterogeneity, including scanner vendor and reconstruction variability for CT-based applications, device and sampling-rate/filter differences for ECG pipelines, and acquisition quality and operator dependence in echocardiography. Such variability makes generalizability and sustained assurance key practical concerns.

At the same time, the transition from model development to safe clinical practice depends on more than accuracy alone. Reporting and evaluation standards increasingly emphasize transparent definition of intended use and clinical context, careful assessment of calibration along with discrimination, and study designs that reflect real-world human-AI interaction and workflow constraints (4–6). When AI outputs drive thresholds or subsequent actions (e.g., referral, additional imaging, initiation of treatment), evaluation should go beyond statistical performance to encompass clinical benefit and decision outcomes, including net benefit-oriented approaches (7). These expectations drive the need for integrated, lifecycle-oriented approaches that link evidence before release, controlled software changes, and post-release monitoring.

Several developments motivate a dedicated synthesis focused on AI-powered cardiovascular devices. First, the field has moved beyond the era where discrimination measures alone were considered sufficient for confidence in the deployment. Methodological studies have highlighted calibration as a frequent point of failure in predictive models, particularly during the external validation phase, arguing that miscalibration can be clinically misleading even when discrimination appears strong (4). Second, real-world performance is dynamic. The dataset shifts, and evolving application models can distort or alter model behavior over time. These require explicit strategies for detection and response rather than the assumption that “once validated, always valid” (2, 3). Third, clinical practice increasingly demands structured attention to human factors, transparency, and reporting completeness. This is especially important for early-stage clinical assessments and subsequent intervention studies, given that user interaction and workflow alignment can determine both safety and effectiveness (5, 8).

In parallel, regulatory and governance expectations for AI-enabled medical devices are rapidly evolving but remain fragmented across jurisdictions and document families (9). While international organizations such as the IMDRF have established common principles through the Software as Medical Device (SaMD) clinical evaluation framework, provide, region-specific approaches differ in terminology, procedural mechanisms, and operational emphasis. These include FDA guidance on predetermined change control planning, EU MDR/IVDR obligations and evolving interaction with the EU AI regulatory framework, and the UK MHRA's software and AI change program (10–12). For cardiovascular programs operating across vendors and sites, this fragmentation creates a practical translation problem. The institutions must translate high-level requirements into viable local procedures for procurement, deployment, version control, and continuous monitoring.

This review presents a lifecycle playbook that integrates peer-reviewed methodological standards with international SaMD principles and region-specific regulatory expectations, focusing on implementation and governance in cardiovascular care.

Governance approaches involve trade-offs and unresolved debates. For example, locked models simplify evidence interpretation, while controlled updates may be necessary to maintain performance under dataset shift. Organizations must also balance the benefits of detailed monitoring with feasibility and resource constraints. By explicitly relating these issues to the realities of lifecycle management and practice in cardiology, this review aims to provide a coherent perspective that is both scientifically grounded and operationally interpretable.

The remainder of the review follows the device lifecycle, addressing evidence generation, change control, and post-market assurance, illustrated with modality-specific considerations for CT-based applications, ECG analysis, and echocardiography.

**Figure 1:**
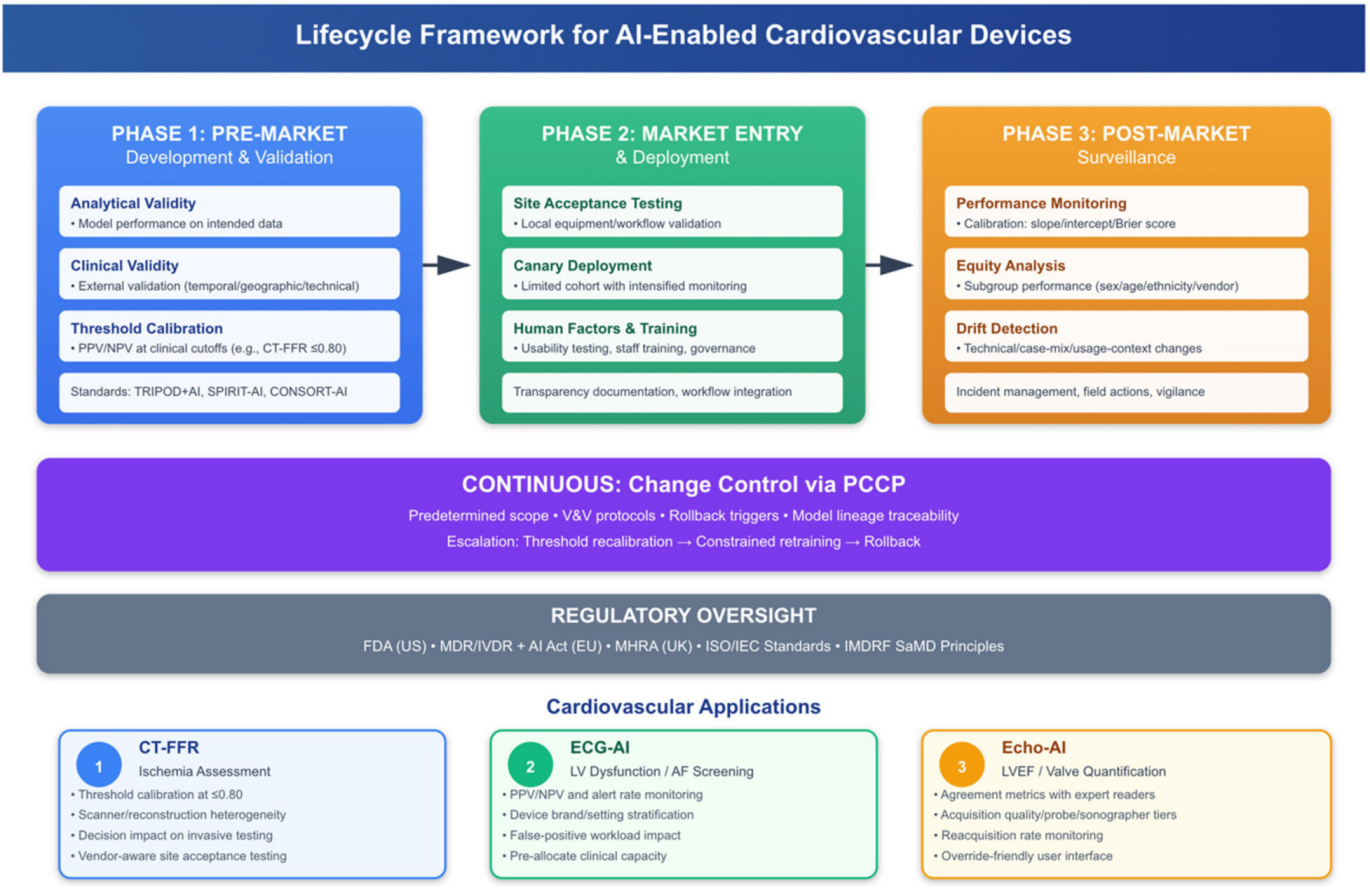
Lifecycle framework for AI-powered cardiovascular devices. Overview of pre-market development and validation, market entry and distribution, and post-market surveillance, supported by continuous change control through predefined change control planning (PCCP), regulatory oversight, and modality-specific implementation considerations.

Figure 1 provides an overview of the lifecycle playbook framework. The pre-market phase focuses on analytical and clinical validation and robust external validation across temporally, geographically, and technically diverse contexts, as well as beyond-discrimination assessment at clinically relevant thresholds. Subsequent phases focus on structured change control and post-market assurance through continuous performance monitoring and predefined escalation pathways.

## 2 Methods

### 2.1 Review design and scope

This article is a narrative review with structured regulatory document analysis focusing on the clinical lifecycle of AI-powered cardiovascular devices. The scope addresses three interconnected domains that determine real-world safety and efficacy: evidence generation and clinical evaluation before deployment, software change management and version control after release, and performance monitoring under real-world heterogeneity and dataset shift.

We focused on cardiovascular applications because these represent mature and rapidly expanding AI use cases with high clinical impact and well-characterized sources of technical and workflow variability. To ground the discussion in practical application realities, we used three representative modalities that differ in acquisition conditions and downstream decision outcomes: CT-derived functional assessment (e.g., CT-FFR use cases), ECG-based analysis (screening/triage), and echocardiography quantification.

### 2.2 Resources and information acquisition

We drew on two complementary evidence streams. The first comprises peer-reviewed scientific literature on clinical evaluation, dataset drift, calibration, decision analytics assessment, early-stage clinical trials, and reporting standards for AI in healthcare. The second encompasses regulatory and standards-based documents that define or strongly shape expectations for AI-powered medical devices and SaMD.

We prioritized primary sources from jurisdictions most influential in the proliferation of cardiovascular devices (United States, European Union, and United Kingdom) and from international organizations informing these systems. Where applicable, we also referenced widely adopted standards addressing software lifecycle processes, usability engineering, and risk management functional. Because this review is translational and cross-jurisdictional, our aim was not to catalog every document or regulation in detail, but to extract recurring expectations that guide practical institutional practice.

Documents were identified through targeted searches conducted between January 2015 and January 2026 across PubMed, regulatory authority websites (FDA, EMA/MDCG, MHRA), and international standards repositories (IMDRF, ISO, IEC). Priority was given to primary regulatory guidelines, harmonization documents, and peer-reviewed methodological studies relating to validation, calibration, dataset change, lifecycle management, and AI application. Documents were included if they contained explicit lifecycle, update management, or post-market surveillance provisions applicable to cardiovascular AI. The extraction process focused on common themes across different jurisdictions (evidence expectations, change management, oversight obligations, transparency, and traceability).

### 2.3 Regulatory document analysis and framework synthesis

For each jurisdiction, we reviewed documents addressing the following topics: clinical evaluation and evidence standards, software change control and update management, post-market surveillance and real-world performance monitoring, transparency and human factors, cybersecurity/traceability. Where applicable, we also reviewed documents on equity or subgroup performance expectations. We then placed the extracted expectations into a comparative lifecycle framework spanning around three phases, including pre-deployment assessment, controlled change during deployment, and post-deployment monitoring. Cross-cutting themes applicable to all phases (intended use and transparency, usability/human factors, traceability and cybersecurity, and subgroup performance monitoring) were also incorporated into this framework.

We clearly distinguished between convergent expectations shared across jurisdictions (e.g., external validation beyond internal performance; lifecycle management; monitoring obligations) and regionally differing implementation mechanisms (e.g., terminology and procedural structures used to manage updates and post-market evidence generation).

This synthesis is summarized through a comparative analysis of regulatory expectations and functionalized through a lifecycle narrative that links evidence requirements, change control, and post-market monitoring to institutional governance tasks.

### 2.4 Development of operational artifacts

The synthesis was operationalized into five illustrative implementation artifacts, presented as conceptual templates rather than validated instruments. These include a clinician-facing transparency template, a site acceptance test (SAT) protocol for local verification, a predefined change control planning (PCCP)-aligned workbook concept to structure update boundaries and verification/validation (V/V) activities, a post-deployment monitoring specification linking key performance indicators (KPIs) to predefined escalation actions, and a RACI (Responsible, Accountable, Consulted, and Informed)-style accountability structure to clarify institutional and vendor responsibilities.

## 3 Foundations and global principles for SaMD

### 3.1 SaMD clinical evaluation: fundamentals shared across jurisdictions

Across jurisdictions, AI-enabled cardiovascular products regulated as SaMD are generally evaluated through dimensions: analytical validity, clinical validity, and clinical utility. Analytical validity addresses whether the software reliably performs its intended technical function. Clinical validity concerns whether the output meaningfully reflects the target clinical condition or outcome. Clinical utility assesses whether the use of the output improves clinical decision-making or patient outcomes compared to standard care. This tripartite framework is consistently articulated in international regulatory convergence efforts and underpins territory-specific requirements in the United States (US), the European Union (EU), and the United Kingdom (UK) (10, 12, 13).

In cardiovascular medicine, these principles must be interpreted against significant application heterogeneity. Differences in scanners, acquisition protocols, device hardware, labeling practices, and workflow integration can lead to dataset shifts between development and real-world use, with consequent changes in model behavior (1–3). This heterogeneity is inherent to many cardiovascular AI applications: CT-based systems are dependent on the scanner manufacturer and reconfiguration processes; ECG-based systems vary depending on the device, sampling rate, and care environment; and echocardiography AI is particularly sensitive to acquisition quality, sonographer technique, and manufacturer-specific image characteristics. Consequently, external validation is increasingly expected to reflect not only geographical separation but also technical and workflow variability relevant to the intended clinical context. A second common principle is acceptance that clinical assessment is lifecycle-based rather than static. Even locked models can experience performance divergence as populations and applications evolve, while adaptive systems clearly change over time. Therefore, regulatory and methodological guidelines converge on the need for governance mechanisms that define what can change, how changes are verified and validated, and how post-implementation performance is monitored and addressed (10–12).

### 3.2 Evidence measures: moving beyond discrimination

A recurring theme in the medical AI literature is that discrimination alone is insufficient for clinical assurance. Models may exhibit high discrimination while producing poorly calibrated probabilities or unstable performance at clinically relevant operating points when applied to novel settings (4). This limitation is particularly important in cardiovascular care, where AI outputs are often interpreted as risk predictions or used to trigger downstream diagnostic or therapeutic actions. Accordingly, contemporary assessment increasingly emphasizes a complementary set of performance dimensions appropriate to the intended use. Discrimination measures such as the area under the receiver operating characteristic curve (AUROC) or the area under the precision-recall curve (AUPRC) remain important, but should be interpreted in conjunction with calibration measures, including calibration slope and intercept, supported by graphical assessment (4). The observed-to-expected ratio (O:E) provides an additional summary of the overall calibration-in-the-large, indicating whether the predictions systematically over- or underestimate event rates (14). Overall probabilistic accuracy can be summarized using measures such as the Brier score, which integrates both discrimination and calibration aspects. When AI outputs are operationalized through explicit thresholds, evaluation should be extended to threshold-dependent metrics such as positive and negative predictive values, and decision analytics approaches that quantify clinical outcomes, including net benefit (7). Comparing predicted and observed outcomes at the operational threshold can provide a practical check of the fit between model outputs and real-world event rates, especially after deployment in new populations.

The same considerations apply to post-market monitoring. Focusing on a single headline metric risk missing clinically significant impairment. Instead, monitoring programs benefit from tracking a small set of interpretable metrics encompassing discrimination, calibration, and threshold performance, interpreted in the context of changes in case mix, acquisition conditions, and clinical workflows common in cardiovascular practice (2, 3).

### 3.3 Reporting frameworks and assurance elements

Reporting standards have been developed to increase the transparency and reproducibility of clinical AI evidence. For predictive models, the TRIPOD-AI statement provides guidance on reporting development and validation, including external validation and calibration assessment (6). For early-stage evaluation of AI decision support systems in real clinical settings, DECIDE-AI emphasizes the identification of the environment, participants, workflow integration, human-AI interaction, and safety oversight (5). When AI systems are evaluated within intervention study designs, CONSORT-AI expands trial reporting standards to address AI-specific considerations (8). Beyond academic reporting, real-world application requires assurance elements that make these principles functional. Across various jurisdictions, expectations are increasingly including transparency documentation for clinicians that explains intended use and limitations, traceability mechanisms that support auditability across software versions, usability evidence demonstrating safe interaction under realistic conditions, structured approaches to software change management, and predefined post-market surveillance plans (10, 12, 13). These elements provide ongoing assurance of pre-deployment evidence and a practical mechanism for managing AI-powered cardiovascular devices throughout their clinical lifecycle.

## 4 Regional frameworks for AI-enabled cardiovascular devices

While regulatory expectations for AI-enabled cardiovascular devices share common foundations, they vary across different jurisdictions in terms of terminology, documentation pathways, and operational emphasis. In practice, these differences are most significant at three points in the lifecycle: (i) what is expected as proof of market entry (including external validation and labeling), (ii) how software changes are managed after the market release, and (iii) how post-market surveillance and real-world performance monitoring are operationalized. The following sections outline the most influential frameworks shaping cardiovascular AI implementation in the US, EU, and UK, based on international SaMD principles (10).

### 4.1 United States: food and drug administration (FDA)

In the US, AI-powered cardiovascular software functions are generally regulated as medical devices via 510(k) or *de novo* pathways, depending on the intended use and risk classification. Consistent with IMDRF principles, FDA expectations emphasize a clearly defined intended use, evidence supporting safety and efficacy for the population and clinical setting, and adequate labeling supporting appropriate clinical interpretation (10).

A recent emphasis is on lifecycle management for models that may be updated after release to the market. The FDA's guidance on Predetermined Change Control Plans (PCCPs) offers a structured approach to iterative software changes while maintaining reasonable assurance of safety and efficacy (13). In practical terms, this framework reinforces three operational expectations that are particularly relevant to cardiovascular AI: clear update limits specifying which aspects of the model or algorithm may change, validation and validation activities associated with each change category, and linkage between planned updates and post-market performance monitoring, with upgrade criteria triggered when real-world performance deviates from expectations (11).

For clinical services adopting these tools, the PCCP approach is naturally compatible with phased deployment practices such as limited initial deployments and local readiness checks, especially where performance depends on site-specific data collection hardware or workflow integration.

### 4.2 European union: MDR/IVDR and artificial intelligence act

In the EU, most AI-assisted cardiovascular software functions fall under the Medical Devices Regulation (MDR), which defines requirements for clinical assessment, risk management, technical documentation, and post-market surveillance (15). The MDR is explicitly lifecycle-oriented: manufacturers must provide continuous post-market surveillance (PMS) and, where appropriate, post-market clinical follow-up (PMCF) proportionate to device risk (15). The practical implication for cardiovascular AI is that expectations of evidence and monitoring are not confined to a pre-market dossier; they must be maintained through systematic post-market activities and surveillance processes.

The EU Artificial Intelligence Act (AI Act) introduces additional AI-specific obligations that complement the MDR/IVDR requirements for medical devices, addressing risks to health, safety, and fundamental rights (16). For AI used in medical devices, the significance of the AI Act is often operational rather than focused on clinical evaluation per se: it reinforces expectations of risk management, data governance, technical documentation, record keeping, transparency, and human oversight in ways that intersect with MDR quality management and PMS/PMCF activities (16). Recent MDCG guidance addressing the interaction between MDR/IVDR and the AI Act highlights the complementary application of both frameworks for AI systems used for medical purposes and the need to integrate AI Act obligations into existing MDR/IVDR processes (17).

From an institutional perspective, EU practice generally requires careful harmonization of clinical evaluation and performance claims, technical documentation and risk management files, and post-market surveillance outputs. Monitoring artifacts can thus support both MDR PMS/PMCF expectations and, where applicable, registration and auditing compliant with the AI Act (15–17). This is particularly important for multi-site cardiovascular applications where device performance may be sensitive to site-level imaging hardware, acquisition protocols and local case mix.

While the MDR and the AI Act are complementary in intent, tensions may arise in practice due to the documentation burden, the overlap between PMS/PMCF and AI Act registration obligations, and potential ambiguity in the distribution of responsibilities between manufacturers and implementers. Hospitals may encounter uncertainty as to whether certain monitoring outputs constitute regulatory reporting or internal management documentation. These areas continue to evolve operationally and may require clarification through future MDCG guidelines and national authority comments.

### 4.3 United Kingdom: MHRA and software as a medical device/AI change programme

In the UK, the MHRA has established a Software as a Medical Device and AI Change Programme aimed at strengthening regulation throughout the software lifecycle and supporting responsible innovation while maintaining patient protection (12). While many core concepts overlap with international SaMD principles, the UK approach places a distinct emphasis on practical, proportional regulation and learning after implementation, including mechanisms supporting iterative improvement and evidence generation in real-world settings (12). For cardiovascular services, a recurring operational theme for the UK is implementation within NHS-like workflows where governance needs to address accessibility, interoperability, cybersecurity, and monitoring in environments with strong operational constraints. In this context, expectations for controlled software change and post-market proof generation can be seen as closely linked: update governance should specify what to monitor after release and how signals (e.g., performance deviation, workflow disruption, security incidents) will trigger proportionate responses (12). This reinforces the practical importance of implementation artifacts that clarify responsibilities across vendors, clinical leaders, and digital/IT governance structures.

### 4.4 Cross-jurisdiction convergence: expected effectiveness in practice

Despite differences in mechanism and terminology, the following convergent expectations are becoming increasingly prominent across the US/EU/UK:

1. Evidence generalizable to real-world heterogeneity. There is increasing emphasis on evidence reflecting the intended clinical context across systems and on performance being assessed beyond internal validation, particularly where devices are deployed in heterogeneous locations (10, 15).
2. Lifecycle governance of software changes. Software updates are treated as a security-related feature requiring risk-proportional structure, traceability, and V/V. While the FDA's PCCP framework provides a clear framework for update planning, the EU and UK approaches place change management within the broader quality management and lifecycle oversight framework (12, 13, 15).
3. Transparency, documentation, and auditability. Across jurisdictions, safe use depends on technical documentation, record-keeping, and traceability that aligns with device risk and regulatory expectations, as well as clear intended use, limitations, and user-facing information (10, 15, 16).
4. Meaningful monitoring and actionability with post-market surveillance. The ultimate goal of governance is not just data collection, but decision-making: monitoring outputs should support the timely identification of deterioration or risk signals and trigger predefined actions such as intensified review, corrective changes within approved limits, or recall/withdrawal where necessary (11, 15, 17).

While regulatory language and legal frameworks differ across jurisdictions, expectations regarding lifecycle assurance of AI-powered cardiovascular devices converge significantly. Table 1 provides a comparative overview of the key requirements shaping clinical evaluation, software change management, and post-market surveillance across major jurisdictions. It summarizes convergent and divergent expectations shaping clinical evaluation, software change management, and post-market surveillance across the US, EU, and UK. The table highlights practical implications for cardiology programs, including documentation needs (transparency and traceability), update management mechanisms, and monitoring elements supporting actionable oversight in heterogeneous clinical settings.

**Table 1:** Comparative overview of lifecycle expectations for AI-powered cardiovascular devices across major jurisdictions.

Topic	United States (FDA)	European Union (MDR/IVDR + AI Act)	United Kingdom (MHRA)	Practical implications for cardiology
Clinical evaluation and Performance evidence	Analytical validity, clinical validity; clinical benefit when claims imply a change in care; external validation awaited (temporal/geographic/technical).	Clinical evaluation under the PMCF and MDR/IVDR; evidence must reflect the intended context; the AI Act covers data management, recordkeeping, transparency, and human oversight.	Evidence aligns with the UK SaMD/AIaMD change program; external validation and usability relevant to the NHS are emphasized.	Validation across scanner/ECG/echo vendor variation; report calibration (slope/intercept/Brier) and threshold level PPV/NPV and decision impact.
Change control	PCCP defines the Scope, V/V protocols, SAT, canary deployment, acceptance bands, and rollback triggers linked to prespecified KPIs.	Controlled updates within the QMS ensuring MDR PMS/PMCF and AI-Act tasks are met.	Change program expects controlled updates with traceability, site-level checks, and escalation actions; AI Airlock enables early intervention.	Implement the versioned PCCP workbook: change log → local SAT → canary cohort → KPI monitoring → action decision (recalibration/constrained retraining/rollback). Update the transparency file with each version.
Post-market audit	Real-world performance monitoring linked to PCCP actions; vigilance; version lineage.	PMS/PMCF + AI-Act recording/monitoring; vigilance via EUDAMED.	NHS-compliant monitoring; event learning from overrides/near-misses.	A single dashboard providing regulatory reporting and local governance.
Transparency and human factors	GMLP + transparency principles; summative usability with representative users.	AI-Act transparency to users; usability in MDR technical documentation.	Transparency/availability emphasized; alignment with NHS workflow.	Provide a user-facing transparency file and show summative usability outcomes with override behavior.
Security, privacy, traceability	SBOM, signed updates, audit logs; inferred provenance; risk-based rollback for security issues.	Security within QMS; AI-Act logging; auditability.	Similar expectations; integration with hospital cyber processes.	Version-source cards; clinical risk-based incident response.
Equity and subgroup performance	Increasing expectations for subgroup analyses in monitoring.	Explicit attention to representativeness/bias; documentation of mitigation measures.	Similar emphasis; equity in the NHS context.	Prespecify fairness KPIs and minimum N; publish an equity appendix every cycle.
Evaluation	External validation; calibration; threshold metrics	Generalizability; metric quartet; PMCF	Proportionate evidence; compliance with NHS workflow	Report calibration (slope/intercept, Brier) along with discrimination and threshold metrics [PPV/NPV, observed-to-expected (O:E) ratio]. Stratify by manufacturer/device and image quality.
Cardiology priorities	CT-FFR: vendor/core panels, threshold calibration at 0.80	CT-FFR: specify vendor/algorithm limits in IFU	CT-FFR: triage pathways	Perform SAT on local equipment before go-live. Stratify monitoring by device vendor/model and maintenance setting. Display threshold-specific metrics (PPV/NPV, O:E on operational outage) on dashboards. Pre-allocate capacity for false-positive workload during canary phases.
			ECG-AI: screening clinic capacity	
	ECG-AI: alert rates, downstream workload	ECG-AI: alert rates bounds, PPV/NPV bands	Echo-AI: acquisition protocols	
	Echo-AI: alignment across quality strata	Echo-AI: quality management, override design		

## 5 AI-specific governance challenges and what reviewers expect to see

### 5.1 Transparency, human factors, and representation

Cardiovascular AI devices are used in workflows under time pressure where misunderstanding of intended use, unclear limitations, or poorly designed interfaces can rapidly lead to patient harm. As a result, regulators and standards organizations are increasingly treating transparency and human factors as essential safety components rather than optional features. International guiding principles on transparency for machine learning-powered medical devices emphasize communicating intended use, performance expectations, limitations, and the role of the human user in a way that supports safe real-world operation (18). In parallel, usability engineering standards for medical devices provide a structured process for identifying and mitigating use-related risks (19).

As discussed in Section 3.2, dataset shifts are expected in real-world cardiovascular AI applications, requiring pre-defined monitoring at both clinical and technical levels (1–3).

Transparency for cardiovascular AI should be function as a clinical safety checkpoint. A transparency document (and associated usage instructions) for clinicians should specify the intended clinical task, inputs and outputs, target population, exclusions, known modes of failure, and how the outputs should and should not be used, and the release history should be communicated to end-users (18). Evidence of usability should demonstrate that representative users can accurately interpret the outputs, recover from errors, and safely intervene in or rely on clinical judgment under realistic time constraints (19). In addition to technical performance institutions may track clinician-centered implementation metrics such as perceived workload (e.g., NASA-TLX), override frequency, alert fatigue indicators, and user satisfaction or burnout-related survey items to ensure that deployment improves care without unintended workflow burden.

### 5.2 Data quality, shortcut learning, and bias: why subgroup assurance isn't optional?

Concerns about bias in medical AI are not theoretical. A widely cited example shows that a commercial algorithm used to allocate care resources exhibited significant regional or social bias because it used healthcare cost instead of healthcare need, thus underestimating disease burden resource-constrained areas or among Black patients with similar risk scores (20, 21). Even if the mechanism remains unclear, evidence that deep learning models can also extract race from medical images highlights those sensitive features can be embedded in data in a way that can create undesirable disparities if not explicitly audited (22).

Subgroup assurance. For cardiovascular AI devices, this should include both clinical layers (e.g., age/gender, comorbidities, disease prevalence) and technical layers (e.g., scanner manufacturer, ECG device type, echocardiography image quality level). Since many cardiovascular devices are used in multiple sites and by multiple vendors, technical subgroup monitoring can be as important as demographic subgroup monitoring to detect significant deterioration. The key for evaluators is not that every subgroup analysis is perfect, but that subgroup performance monitoring is prespecified, interpretable, and actionable when significant deviations emerge (18).

Operational implication. Subgroup monitoring should be built into the post-market plan from the start. It should meet the minimum sample size expectations for stable estimates and a defined response (e.g., targeted investigation, recalibration, constrained retraining within update governance boundaries, or rollback) when gaps exceed predefined tolerances. This approach aligns bias concerns with lifecycle management rather than considering equity after the fact (21, 22).

Although hallucinations are more commonly discussed in generative AI systems, clinically used predictive or diagnostic models may exhibit output instability or irrational results when confronted with out-of-distribution inputs. Monitoring unexpected confidence patterns, abrupt shifts in output distributions, or clustering of clinician interventions can function as indirect detection signals.

### 5.3 Continuous learning, drift, and change governance

Even when models are not designed to be continuously updated, deployment environments evolve. Dataset drift can result from changes in the patient mix, workflow, acquisition hardware, software updates in upstream systems, or protocol changes. Methodological studies have highlighted that dataset drift is an expected feature of real-world clinical AI and should be explicitly managed rather than discovered by chance after failures occur (2, 3). This is one reason why regulators are moving towards lifecycle thinking, including structured management of updates, V/V expectations, and post-deployment monitoring based on predefined actions (11, 12).

From an assurance standpoint, drift management is not just a statistical problem but also a governance problem: Organizations need clarity on which changes are allowed, how changes will be tested before release, and which monitoring signals will trigger upgrades. This logic is central to lifecycle change governance frameworks that pre-define what can change, how updates will be verified/approved, and how monitoring will send notifications to a higher level. Structured lifecycle change governance frameworks provide operational mechanisms to predefine what might change, how updates will be verified and approved, and how monitoring signals will trigger proportionate responses. The FDA's PCCP offers a clear statement, while similar expectations are found in EU MDR quality management systems and the UK SaMD/AI change program (13).

Operational implication. Reviewers are increasingly looking for a consistent link between (i) pre-deployment evidence, (ii) change governance (including what might change and how it is tested), and (iii) post-deployment monitoring that triggers predefined actions (11, 12). In cardiovascular workflows, this typically requires local field controls (e.g., field acceptance testing) and phased deployments.

### 5.4 Cybersecurity, traceability and auditability

Since AI-enabled cardiovascular devices are software-intensive and often networked, cybersecurity is integral to overall device safety. The FDA's cybersecurity guidance emphasizes cybersecurity as part of device safety and quality system considerations, including documentation and risk-focused controls; it also highlights the importance of software component transparency (including a software bill of materials in a cybersecurity context) and vulnerability management processes (23). These expectations are consistent with broader software lifecycle and risk management standards widely used in device auditing, including risk management processes throughout the device lifecycle (24) and software lifecycle processes for medical device software (25).

Operational implication. Traceability for AI-enabled cardiovascular devices should encompass not only the software version but also the associated configuration, model lineage, and deployment environment dependencies, and include auditable records appropriate to clinical risk. Cybersecurity monitoring and vulnerability management should be integrated into the same governance pathways as performance monitoring, as security incidents may require urgent response (23–25).

## 6 Implementation and governance: requirements for hospitals to operate safely

Regulatory principles only become clinically meaningful when translated into repeatable organizational processes. In practice, hospitals implementing AI-powered cardiovascular devices need a small set of standardized elements supporting procurement, commissioning readiness, controlled updates, and ongoing monitoring. Several expectations are repeated across various jurisdictions: clear intended use and transparency to users, usability engineering, traceability and risk management, structured change governance, and actionable post-market monitoring (10, 11, 18, 19, 24). The operational elements presented are conceptual, non-proprietary templates for adaptation; no commercial tools or services are being promoted.

### 6.1 Transparency documentation (user-facing and submission-grade)

A practical starting point is a clinician-facing transparency document (“transparency card” or “transparency file”) that provides guidance on intended use, input/output, target population, exclusions, known limitations, and safe interpretation. For AI-powered software, transparency also includes version history and update impacts so clinicians understand whether model behavior will change over time. These expectations are consistent with international transparency guidelines jointly developed by the FDA, Health Canada, and MHRA (18).

Implementation note. Hospitals benefit from maintaining two consistent versions: a short, user-interface-accessible clinician card (for daily use) and a longer governance/procurement document that archives update notes and key validation summaries.

### 6.2 Site acceptance testing (SAT) before go-live

Hospitals should treat site go-live as a safety-critical transition rather than an IT deployment step. Site acceptance testing (SAT) is the local verification that the system performs as expected on site-specific data paths, devices, and workflows. SAT typically includes the following: First, end-to-end input/output controls; second, small local verification of performance and calibration suitable for intended use (especially where thresholds trigger action); and third, a workflow rehearsal verifying those clinicians can correctly interpret outputs and safely override or postpone them as needed. SAT is particularly important in cardiovascular AI because heterogeneity is often technical (scanner and reconstruction variability, ECG device filters/sampling rates, and echocardiography acquisition quality). Usability engineering standards reinforce the need to identify and mitigate risks associated with use under realistic conditions (19), and risk management standards support documenting hazards, controls, and remaining risk throughout the lifecycle (24).

Implementation note: SAT results should be recorded in a standardized template and linked to governance sign-off (e.g., “go/no-go”), with predefined corrective steps when minimum criteria are not met.

### 6.3 Change governance and controlled rollout (including predefining change planning)

A central operational challenge is that software changes (whether bug fixes, user interface adjustments, model recalibration, or retraining) can alter clinical behavior. The FDA's guidance on PCCPs formalizes a forward-looking approach: it defines planned and permitted changes, how V/V will be performed for each type of change, and how changes relate to post-market monitoring and corrective actions (11).

Even outside the US, the operational logic is similar: hospitals need clarity on what might change, how updates are tested, how version traceability is maintained, and when upgrades (including rollbacks) are necessary. Software lifecycle process expectations for medical device software further strengthen structured development and maintenance processes (25).

Implementation note. Hospitals typically handle this as follows: change log → pre-release V/V testing → local SAT → staged (“canary”) deployment → intensified monitoring → full deployment with predefined rollback triggers (11, 24).

### 6.4 Unified monitoring dashboard (regulatory PMS/PMCF + institutional governance)

Post-deployment assurance requires a monitoring system that serves regulators and quality systems on one hand, and hospital management on the other. A single dashboard is preferred over parallel systems as it reduces duplication and increases accountability.

In practice, the panel content should support longitudinal performance monitoring (including threshold behavior where relevant), subgroup monitoring across clinical and technical layers, workflow, and workflow indicators (e.g., alert rates and downstream workload), and traceability of version/configuration, and environment dependencies.

This approach aligns with the lifecycle emphasis of SaMD clinical assessment (10) and the expectation that update management will be linked to monitoring and actionability (13). Transparency principles further support making monitoring-related information visible to clinical users and management teams, especially when changes affect usage (18).

Implementation note. For management efficiency, hospitals can export a short monthly report (one page per site, one tool per dashboard) from the dashboard summarizing current performance, exposed risks, and actions taken against predefined tolerances.

### 6.5 Accountability and escalation pathways (RACI + incident learning)

Governance fails when an “everyone is responsible” approach because then no one is accountable. A lightweight RACI-style accountability structure clarifies who is responsible for monitoring, who has the authority to take action, who should be consulted for safety and cybersecurity decisions, and who should be informed when updates occur. This structure should integrate clinical safety oversight (covering interpretation, workflow alignment, and override models), data and AI oversight (addressing performance and deviation monitoring), IT and security oversight (including patching, logging, and access control), and vendor liabilities (such as release notes, V/V summaries, and traceability documentation). Risk management standards emphasize the documentation of hazards, controls, and post-market learning throughout the lifecycle (24), while software lifecycle standards support disciplined maintenance processes (25). Transparency guidance also supports consistent communication with users when changes affect device behavior (18).

Implementation note: Escalation pathways should be predefined and proportionate, include review timelines (e.g., rapid review for safety-critical signals), and intensified monitoring should include clear criteria for corrective action or rollback within approved limits (13).

**Figure 2:**
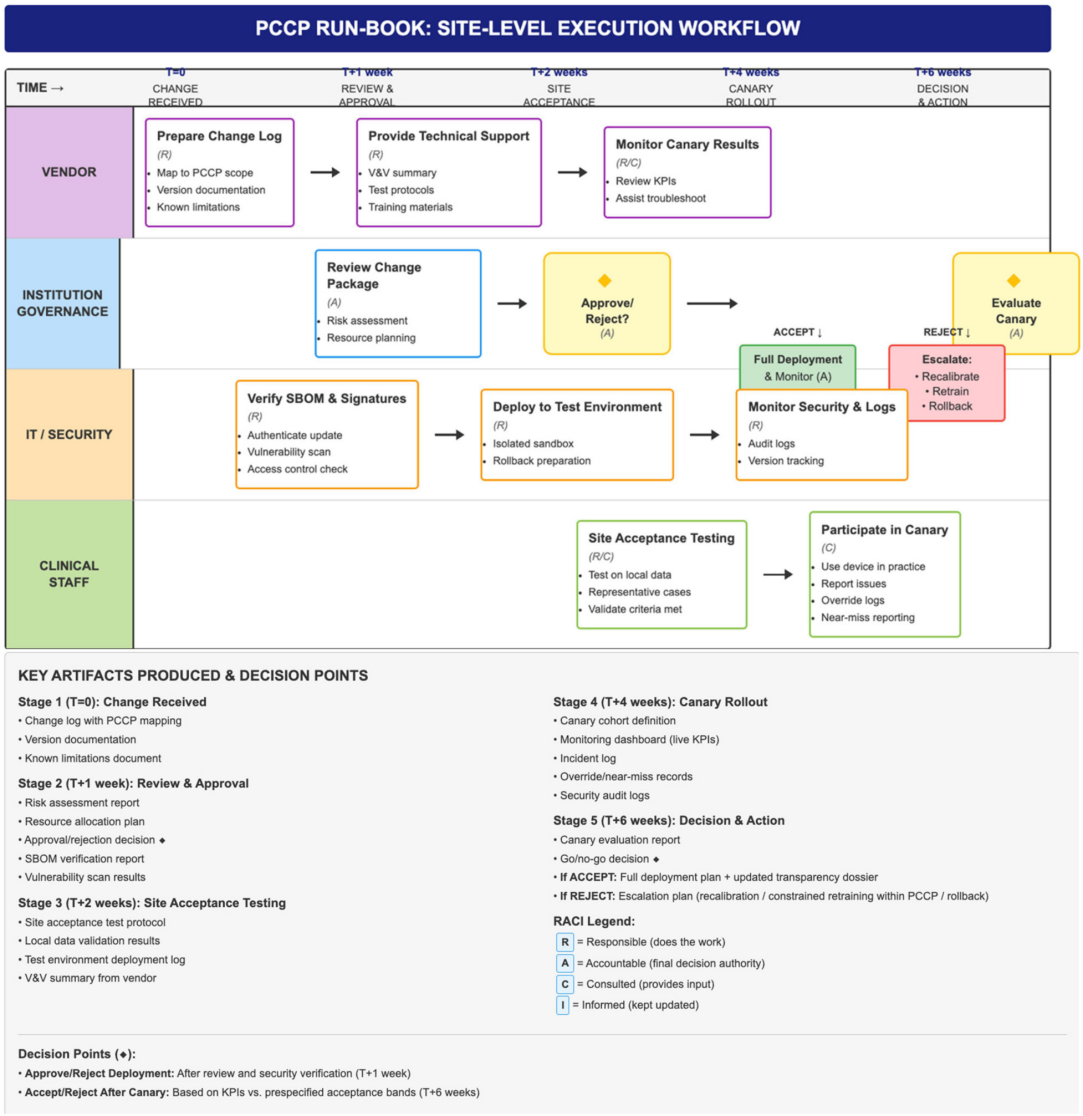
Site-level execution workflow for controlled updates within a PCCP-aligned governance model. Illustrative workflow depicting how AI-enabled device updates can be implemented with versioned documentation, local risk review, site acceptance testing, staged (“canary”) rollout, and predefined go/no-go decision points linked to monitored performance. Roles are summarized using a RACI convention (Responsible, Accountable, Consulted, Informed). This workflow is presented as an example of operationalization and may require adaptation to local institutional policies and jurisdiction-specific requirements. **Notes.** “Canary rollout” refers to limited deployment with intensified monitoring prior to broader release. “Rollback” denotes reversion to a prior version when safety or performance assurance cannot be maintained. PCCP, predetermined change control plan; V/V, verification and validation; RACI, responsible–accountable–consulted–informed.

An illustrative site-level workflow demonstrating how controlled software updates may be operationalized within a PCCP approach is provided in Figure 2.

## 7 Post-market surveillance and monitoring: operating within predefined performance tolerances

Post-market surveillance for AI-enabled cardiovascular devices should be designed to do more than simply “collect data.” Its aim is to detect clinically significant performance changes early, determine whether the changes reflect case mix or a technical change, and trigger proportionate actions that maintain safety and efficacy over time. This lifecycle logic is consistent with the IMDRF SaMD clinical evaluation framework (10) and jurisdiction-specific expectations linking ongoing surveillance to controlled change governance (11, 15).

### 7.1 Surveillance plan and cadence

A practical surveillance plan specifies what is being monitored, how often, in whom, and what happens when signals exceed predefined thresholds. Since dataset shifts are expected in clinical AI applications, surveillance frequency should be adjusted according to clinical risk and volume, and intensified monitoring should be carried out after meaningful updates or during phased (“canary”) deployments (2, 3, 13).

At minimum, surveillance planning should define the performance metrics to be monitored and establish definitions of clinically significant subgroups, including technical layers such as device supplier or protocol, where relevant. The plan should also specify minimum sample sizes for stable estimation and set an appropriate review frequency; monthly for routine monitoring, but more frequent for high-risk tools or post-update canary deployments. Finally, pathways for escalation to investigation and action should be clearly articulated. In the EU context, PMS/PMCF obligations formalize this lifecycle approach for medical devices, emphasizing the ongoing production of evidence proportional to risk (15).

### 7.2 Metrics to monitor: beyond a single headline number

A common mode of failure in management is over-reliance on discrimination. Calibration has been emphasized as a frequent “Achilles' heel” of predictive analytics, especially under external validation and changing deployment conditions (4). Accordingly, post-market monitoring should track a small set of interpretable metrics encompassing discrimination, calibration, and clinically significant operating points.

Consistent with Section 3.2 monitoring track discrimination, calibration, and threshold-dependent metrics rather than relying on a single headline measure (7, 19, 24).

For cardiovascular AI, it is often important to classify monitoring according to technical conditions (e.g., scanner vendor and reconfiguration parameters for CT pipelines, ECG device type and sampling rate, and maintenance environment or echocardiography image quality level), as technical change may be a dominant cause of deviation.

### 7.3 Defining predefined tolerances and actionability

Monitoring is most effective when linked to predefined performance tolerances (acceptance bands) and proportioned escalation actions. Across all jurisdictions, lifecycle governance frameworks expect monitoring outputs to be linked to explicitly predefined V/V pathways and update oversight mechanisms (13).

In practice, tolerances should be justified by the balance between intended use and clinical harm. For example, a small calibration shift may be clinically acceptable for informational tools, but unacceptable for tools that trigger automated escalation, referrals, or treatment changes. Since uncertainty depends on event rate and sample size, tolerances should be interpreted with confidence intervals and minimum sample thresholds for reliable inference.

For example, a CT-FFR tool operating at a threshold of ≤0.80 might define an acceptable range for PPV between 0.75–0.85 based on pre-implementation validation. A drop below 0.72 in ≥50 consecutive cases or two follow-up cycles might trigger a formal investigation, while a drop below 0.70 showing a statistically significant deviation from the baseline (95% confidence interval not coinciding with the previous estimate) may require recalibration or restricted retraining within predefined management limits.

### 7.4 Drift detection and escalation pathways

When monitored performance exceeds predefined tolerances, a structured path should be followed to distinguish calibration drift, broader performance degradation, subgroup-specific deviation, and safety-related signals. As discussed in Section 3.2, dataset drift is expected in real-world cardiovascular AI applications and requires investigation of potential changes in case mix, acquisition conditions, workflow, or recent updates.

Correction should be proportionate, traceable, and consistent with predefined update management limits. Corrective actions may include recalibration, restricted retraining within allowable limits, phased redeployment, or rollback where safety cannot be ensured. Documentation and user communication should be proportionate to the risk and compliant with relevant jurisdictional requirements (13).

### 7.5 Documentation and governance reporting

To prevent monitoring from becoming an unstructured data exercise, institutions should produce concise, standardized governance outputs: periodic summaries of monitored performance against predefined tolerances, marked subgroups and technical layers, measures taken, and open risks. This is consistent with the lifecycle emphasis of SaMD clinical evaluation (10) and the MDR's expectations for post-market documentation for medical devices (15). An example of a unified monitoring dashboard integrating performance, subgroup, operational, and governance-relevant signals is shown in Supplementary Figure S1.

## 8 Modality-specific application considerations in cardiovascular AI

While regulatory principles and lifecycle governance expectations are broadly shared, their practical application varies by modality. Differences in acquisition technology, workflow integration, and clinical decision outcomes shape both validation requirements and post-deployment monitoring priorities. To demonstrate how lifecycle governance can be operationalized in practice, we apply the framework to three representative cardiovascular AI modalities: CT-based functional assessment, ECG-based analysis, and echocardiography quantification.

### 8.1 CT-based functional assessment (e.g., CT-derived FFR use cases)

AI-enabled CT-based functional assessment tools are used to obtain physiological information from coronary CT angiography and often influence subsequent decisions such as referral for invasive angiography or revascularization. These tools exemplify a high-risk diagnostic context where errors can lead to unnecessary invasive procedures or missed clinically significant disease.

A defining challenge for this modality is technical heterogeneity. Model performance can vary depending on factors that differ between institutions, such as scanner manufacturer, detector technology, reconstruction algorithm, acquisition protocol, and image quality. While published evaluations demonstrate strong diagnostic performance in controlled environments, they also highlight the need for careful external validation when tools are used across a variety of scanners and centers (26, 27). Consequently, reviewers and regulators increasingly expect validation datasets to reflect meaningful technical diversity rather than data from a single vendor or a single center.

From a lifecycle perspective, threshold behavior and calibration are particularly important. CT-derived functional outputs are often dichotomized to guide clinical action. Therefore, miscalibration at the operating threshold can directly lead to clinical outcomes. Pre-deployment evaluation should include assessment of calibration and threshold-specific performance, while post-deployment monitoring should track these metrics over time and across technical layers (4). Given the potential impact on invasive procedures, predefined tolerances for drift may be narrow, and low thresholds may be set for escalation and review.

**Implementation summary**. For CT-based functional AI, organizations should prioritize SAT in local scanner configurations, threshold-driven monitoring classified on a vendor/protocol basis, and conservative change governance with staged rollout for substantive updates.

### 8.2 ECG-based AI for screening and triage

AI-powered ECG analysis tools are increasingly used for screening, risk classification, and triage in a variety of settings, including outpatient clinics, emergency departments, and outpatient monitoring. Compared to CT-based tools, ECG AI is typically deployed at large scale and high frequency, often with lower risk per use but potentially significant downside workload impacts.

A key source of variability in ECG AI is device and context heterogeneity. ECGs can be obtained using different manufacturers, sampling rates, filter settings, electrode configurations, and maintenance settings, each of which can affect signal characteristics. External validation studies have shown that performance can vary between these conditions, reinforcing the need for explicit documentation of acquisition characteristics and representative validation (28, 29).

Since many ECG AI tools are used for screening, positive predictive value and alert load are more clinically significant than global discrimination alone. A modest change in calibration or prevalence can significantly impact subsequent referrals and clinician workload. Consequently, post-market monitoring should focus on alert rates, threshold performance, and subgroup behavior in addition to traditional performance metrics (2, 7).

**Implementation summary**. For ECG-based AI, management should focus on calibration and alert rate stability across devices and settings, and monitoring should rely on workflow impact and clearly defined upgrade paths in case of workload or performance deviation.

### 8.3 Echocardiography AI for quantification and interpretation

Echocardiography AI tools are commonly used for automated chamber quantification, functional assessment, and measurement assistance. These systems operate in a highly operator-dependent imaging environment where variability in image quality and technique is an integral part of routine care.

Unlike CT or ECG, echocardiography performance is greatly influenced by sonographer skill, probe positioning, patient body structure, and image quality, which can vary significantly even within a single institution. Studies evaluating echocardiography AI have shown promising accuracy under controlled conditions but have also highlighted sensitivity to image quality and acquisition variability (30, 31). As a result, external validation and monitoring must explicitly account for image quality layers and acquisition conditions.

From a governance perspective, usability and human factors are particularly important. AI outputs are typically presented in real-time along with images and measurements, and misinterpretation or over-reliance can occur if limitations are not clearly communicated. Therefore, transparency documentation and usability testing should highlight instances where outputs are unreliable or should be disregarded, and include qualitative signals such as post-market monitoring, override frequency, and user feedback (18, 19).

**Implementation summary**. For echocardiography AI, organizations should prioritize image quality-sensitive validation and monitoring, robust usability assessment, and conservative interpretation of performance metrics that may mask quality-related failure modes.

### 8.4 Cross-modality lessons

Several common lessons emerge across these three modalities. First, external validation should reflect modality-specific sources of heterogeneity, whether technical, contextual, or operator-dependent. Second, monitoring priorities should align not only with statistical performance but also with clinical decision outcomes. Third, change governance and escalation thresholds should be proportionate to risk, with tighter tolerances for tools that directly impact invasive or irreversible decisions. Finally, methodological considerations reinforce the value of an adaptive rather than prescriptive, unified lifecycle framework that allows organizations to tailor practice to local clinical realities while applying common principles.

## 9 Discussion

### 9.1 Principal findings and synthesis

This review synthesizes methodological standards, regulatory frameworks, and implementation considerations relevant to AI-enabled cardiovascular devices across the clinical lifecycle. Across jurisdictions and modalities, several consistent themes emerge. First, evidence expectations extend beyond discrimination to calibration, threshold performance, and decision consequences (4, 7). Second, software change is treated as safety-relevant, motivating predefined governance rather than *ad hoc* reassessment, particularly for AI-enabled device software functions (13). Third, post-market surveillance is expected to be actionable, linking monitoring outputs to predefined responses (10, 15). Finally, while regulatory language varies, expectations converge on lifecycle-based assurance integrating pre-deployment validation, controlled updating, and continuous monitoring in the presence of dataset shift (2, 3).

A central contribution of this review is the translation of these convergent expectations into implementable institutional practices. Rather than proposing new algorithms or regulatory requirements, we map existing standards to operational artifacts (transparency documentation, SAT, structured update governance, unified monitoring dashboards, and accountability frameworks) that support consistent procurement, deployment, and ongoing assurance (18, 19, 24).

### 9.2 Interpretation in the context of previous studies

Many cardiology AI reviews focus on development performance or clinical outcomes, but often treat deployment as a terminal step rather than a dynamic process. In contrast, deployment-oriented literature highlights the importance of management dataset shift and workflow variability in real-world setting. Reporting frameworks reflect this shift: TRIPOD + AI strengthens expectations for transparent reporting of prediction models including calibration and external validation, DECIDE-AI focuses on early-stage clinical evaluation in real settings, and CONSORT-AI extends trial reporting for AI interventions (5, 6, 8). Together, these developments support a lifecycle framing in which evidence generation, update governance, and post-market surveillance are inseparable components of responsible adoption (10, 11, 15).

### 9.3 Debates and unresolved questions

Several debates remain unresolved and require explicit attention.

**Locked and adaptive models**. A persistent debate concerns whether AI medical devices should remain “locked” after approval or be allowed to adapt over time. Locked models offer clearer reproducibility and regulatory interpretability, while adaptive approaches may better mitigate performance drift under dataset shift (2, 3). PCCP represents a pragmatic compromise by allowing limited change with predetermined V/V expectations. However, empirical evidence comparing the long-term safety and efficacy of locked versus adaptively maintained systems remains limited (13).

**What constitutes sufficient evidence?** Evidence thresholds remain contested. Some stakeholders argue that retrospective validation may suffice for low-risk applications, while others emphasize prospective studies and randomized assessments demonstrating clinical impact whenever possible. This debate is sharpened when models are used at explicit operating points that trigger action. In these conditions, calibration and decision analytics assessment can significantly impact clinical outcomes even when discrimination is high (4, 7).

**Monitoring burden, feasibility, and equity**. Continuous monitoring increases security but also increases resource demands. This raises a practical equity concern: stringent governance expectations can disproportionately burden smaller or resource-constrained institutions. Equity issues are not hypothetical; leading studies have shown that algorithmic design choices can encode structural inequalities and that sensitive features can be inferred from clinical data even if not explicitly included (21, 22). These facts strengthen the rationale for subgroup-focused assurance approaches while also underlining the need for feasible monitoring designs.

**Explainability and performance**. There is an ongoing debate between the adequacy of *post-hoc* techniques for high-risk decisions and inherently interpretable modeling approaches. Some argue that black-box models may be insufficient for ensuring trust and accountability, advocating for interpretable models whenever possible, while others emphasize performance and rigorous monitoring as primary safety controls (9, 32). This debate is directly relevant in time-pressured, cardiovascular workflows where clinicians need reliable, actionable outcomes without excessive cognitive load.

Implementation requires digital infrastructure capable of providing automated data extraction, version logging, and secure audit trails. Institutions without integrated PACS/EHR interoperability may face significant engineering costs. Workforce demands include clinical AI surveillance leaders, data analysts, and management committees. In resource-constrained environments, tiered monitoring cadences proportional to risk and case volume may be adopted.

Formal cost-effectiveness analyses comparing monitoring intensities remain limited; however, if organizations avoid structured auditing due to resource constraints, the disproportionate management burden can paradoxically reduce safety.

### 9.4 Practical implications for stakeholders

For healthcare organizations, this review supports treating AI-powered cardiovascular devices as part of a clinical quality and safety infrastructure rather than as isolated digital tools. Organizations benefit from standardized transparency documentation and evidence of usability, structured change governance linked to defined monitoring triggers, and clear accountability for escalation and corrective actions (13, 18, 19, 24).

For device developers, the findings highlight the importance of designing products with lifecycle governance in mind. This includes establishing traceability, update documentation, and monitoring mechanisms that support post-market surveillance expectations and institutional quality systems (10, 11, 15). For regulators and policymakers, areas where additional guidance that could reduce uncertainty is needed include quantitative acceptance thresholds, operational expectations for subgroup monitoring, and risk-proportional pathways for adaptive systems. In the EU context, these issues are addressed alongside broader obligations for lifecycle oversight under MDR and the evolving intersection with AI-specific governance tools (15, 16).

Beyond these regions, various regulators converge on similar lifecycle expectations. Four jurisdictions are particularly instructive for cardiovascular AI: Canada offers clear MLMD pre-market expectations and common transparency principles; Australia has mature SaMD reforms and active AI/MDSW guidance; Japan has developed PCCP-like change plan concepts through partial change approvals; and Singapore integrates lifecycle guidance with a national cybersecurity labeling scheme. These are united in three operational demands: task-appropriate transparency for users, traceability and controlled updates with model history, and post-market monitoring demonstrable to both regulators and hospital management. International regulatory convergences outside the US, EU and UK are summarized in Table 2.

**Table 2:** Selected non-US/EU/UK frameworks relevant to AI-enabled software as a medical device (cardiovascular focus).

Jurisdiction	What's unique/new?	What to show reviewers?	Practical note for cardiology	References
Canada (Health Canada)	Co-authored transparency principles with FDA/MHRA (2024) and published pre-market guidance for MLMD (2025).	Labeling/UI transparency, lifecycle transparency, compliance with GMLP.	Mirror the US transparency efforts; include user-facing “how to interpret outputs” information and update notes on dossier.	FDA/HC/MHRA Transparency Principles (2024); Health Canada MLMD Pre-market Guidance (33).
Australia (TGA)	Dedicated AI/MDSW pages (updated in 2025) and consolidated SaMD reforms; reliance on risk-based SaMD changes from 2021.	Confirm device purpose under the TGA's SaMD scope; demonstrate proof of compliance and change controls consistent with QMS.	For CT/ECG/echo AI, emphasize vendor heterogeneity and local site acceptance; language clearly aligns with TGA's SaMD approach.	TGA SaMD/AI web guidance (2025 updates).
Japan (PMDA/MHLW)	PMDA reports highlight change control concepts similar to PCCP and the use of partial change approvals, signaling a move toward ecosystem lifecycle management.	Demonstrate planned update checks and traceability/model lineage; indicate that retraining is within predefined boundaries.	If targeting Japan, pay attention to the translation of PCCP ideas; clearly state safeguards and rollbacks in technical documentation.	PMDA/MHLW notices on program medical devices and partial change approvals (2023–2025).
Singapore (HSA + CSA)	HSA digital health/SaMD lifecycle guide (2024) and national cybersecurity labelling scheme for medical devices (2024).	Evidence of SBOM, signed updates, audit logs; map PMS to HSA's real-world performance expectations.	Section 5, 6 security/traceability controls are already compliant; clearly state these in your Singapore applications.	HSA SaMD lifecycle guidance (2024); CSA CLS-MD materials (2024/2025).

This table is provided as a high-level snapshot and does not constitute legal guidance.

SaMD, software as a medical device; MLMD, machine learning-enabled medical device; SBOM, software bill of materials.

### 9.5 Limitations

This review has several limitations. First, it focuses on three jurisdictions (US, EU, UK) and does not comprehensively address global regulatory approaches. Regulatory guidance and implementation practices remain evolving, and interpretations may change as new documents and real-world experience accumulate (13, 16). Second, the operational artifacts described represent conceptual syntheses derived from methodological standards and regulatory principles rather than prospectively validated implementation tools for the future, and applicability may vary depending on organizational resources and management maturity. Third, we have not formally conducted cost-effectiveness analyses of management and monitoring strategies, which limits conclusions about scalability. Fourth, cardiovascular examples may not be fully generalizable to other clinical setting with different workflows and risk profiles.

Finally, this review follows a structured narrative synthesis rather than a formal systematic review methodology. The literature inclusion process is purposeful and focuses on regulatory frameworks, methodological standards, and lifecycle governance themes related to cardiovascular AI. While this approach supports translational interpretation across different jurisdictions, it may lead to selection bias despite efforts to capture the convergence of international expectations.

### 9.6 Future directions

Future studies should prioritize prospective application studies evaluating lifecycle governance strategies in real-world cardiovascular settings, including resource requirements and impacts on safety and clinical outcomes. Methodological investigations are needed to improve calibration monitoring, deviation detection, and subgroup assurance for adaptive systems under realistic sample size constraints (2, 4). Regulatory science initiatives could explore harmonized acceptance criteria and practical risk-based oversight models for iterative AI updates (13). Finally, health systems research should examine how governance infrastructure can be scaled equitably and how safety-focused requirements do not inadvertently exacerbate inequalities (9, 21, 22).

## 10 Conclusion

This review outlines a lifecycle governance framework for AI-enabled cardiovascular devices that integrates methodological standards for clinical evaluation with current regulatory expectations across major jurisdictions. Rather than treating validation, software updates, and post-market surveillance as isolated activities, the proposed guidance links evidence generation, controlled change management, and continuous assurance to a consistent operational model appropriate to real-world clinical heterogeneity.

By translating regulatory principles into practical implementation artifacts (including transparency documentation, SAT, PCCP workflows, performance monitoring dashboards, and accountability structures), this work provides hospitals and vendors with a concrete foundation for the safe deployment, scalable adoption, and sustainable surveillance of cardiovascular AI systems. The emphasis on calibration, threshold-level performance, subgroup assurance, and release traceability addresses common failure modes observed after deployment and supports clinically meaningful monitoring beyond basic accuracy metrics.

As AI-enabled devices continue to evolve and regulatory frameworks mature, consistent lifecycle governance will be essential to protect trust, safety, and clinical value. The framework presented here supports procurement decisions, institutional governance, and quality assurance activities, while also being adaptable to jurisdiction-specific requirements and emerging standards of evidence.
