# Supplementary material for: AI-enabled cardiovascular devices: a lifecycle playbook for evidence, change control, and post-market assurance
Source: Front Digit Health. 2026 Mar 19;8:1785381. doi: 10.3389/fdgth.2026.1785381 (PMC13044001; doi:10.3389/fdgth.2026.1785381)
Supplement: Supplementary file 1 [file Datasheet1.docx]

| 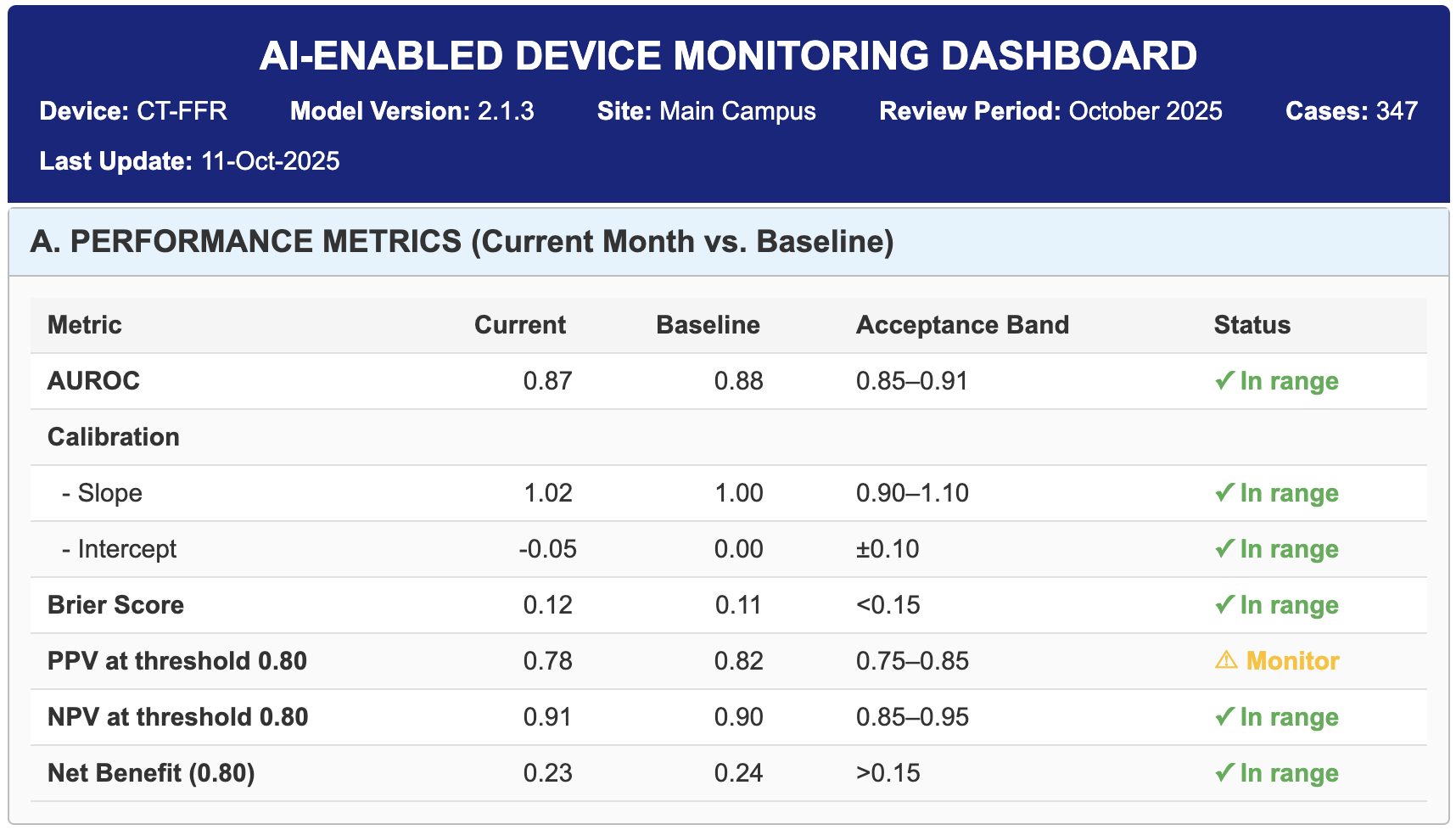 |
| --- |
| 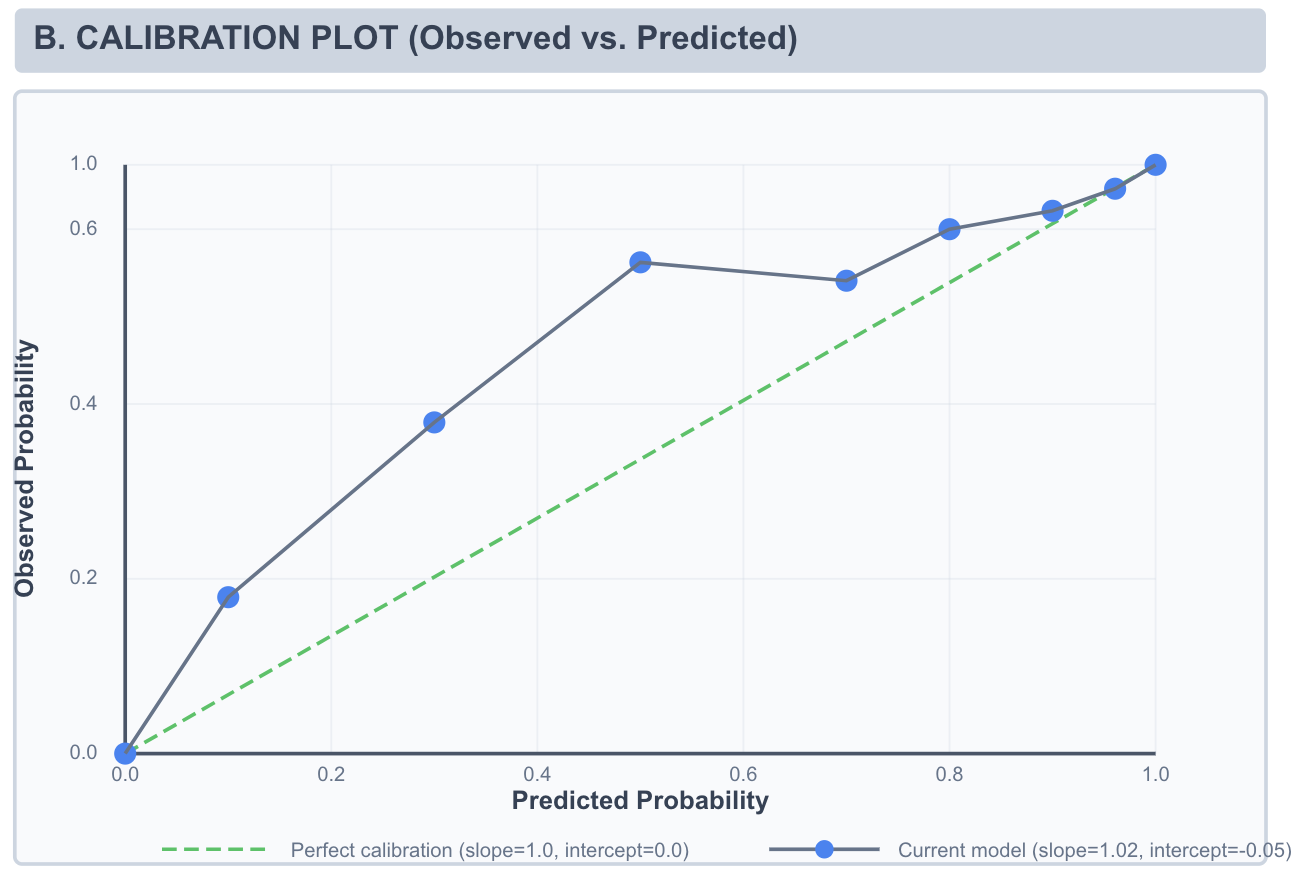 |
| 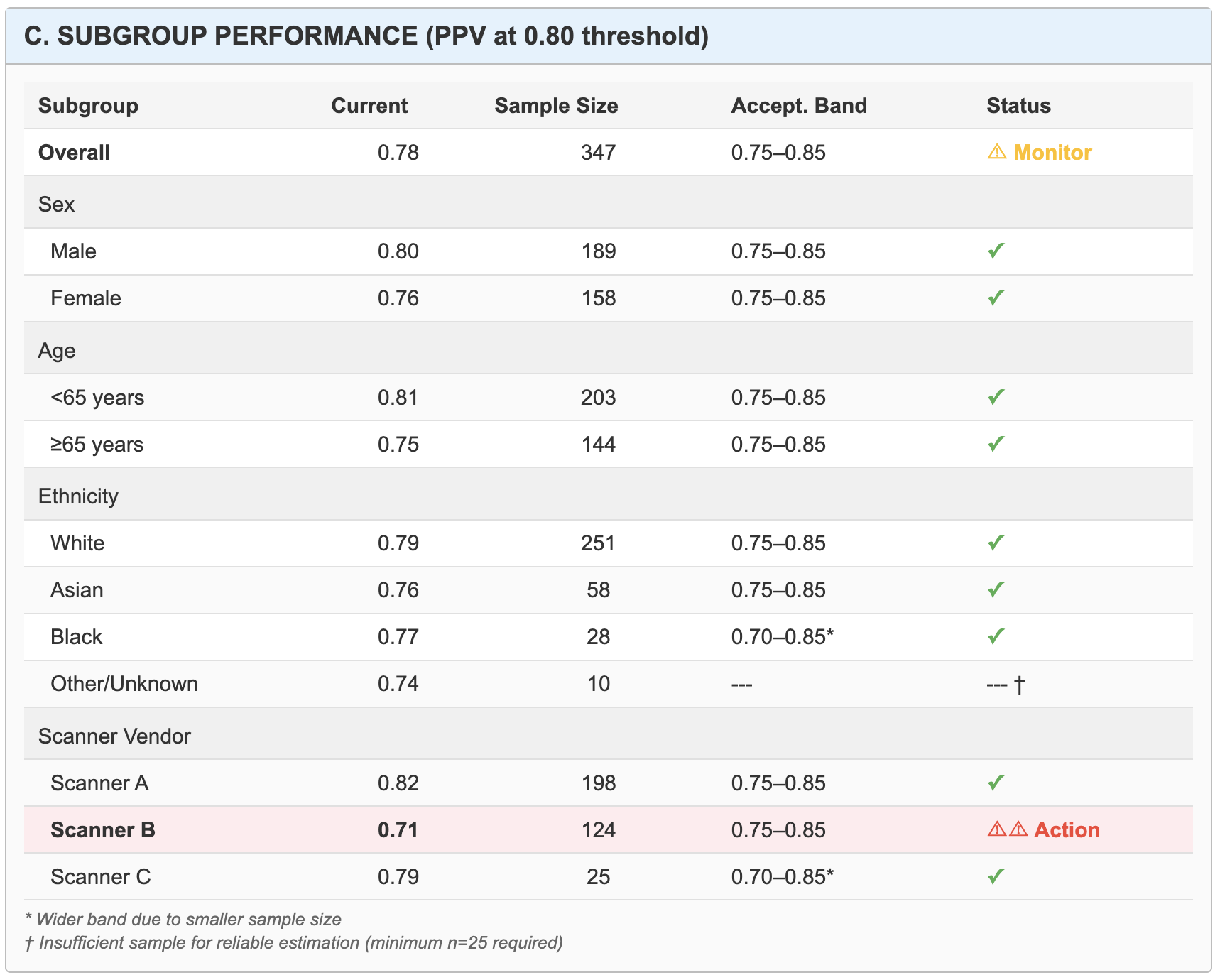 |
| 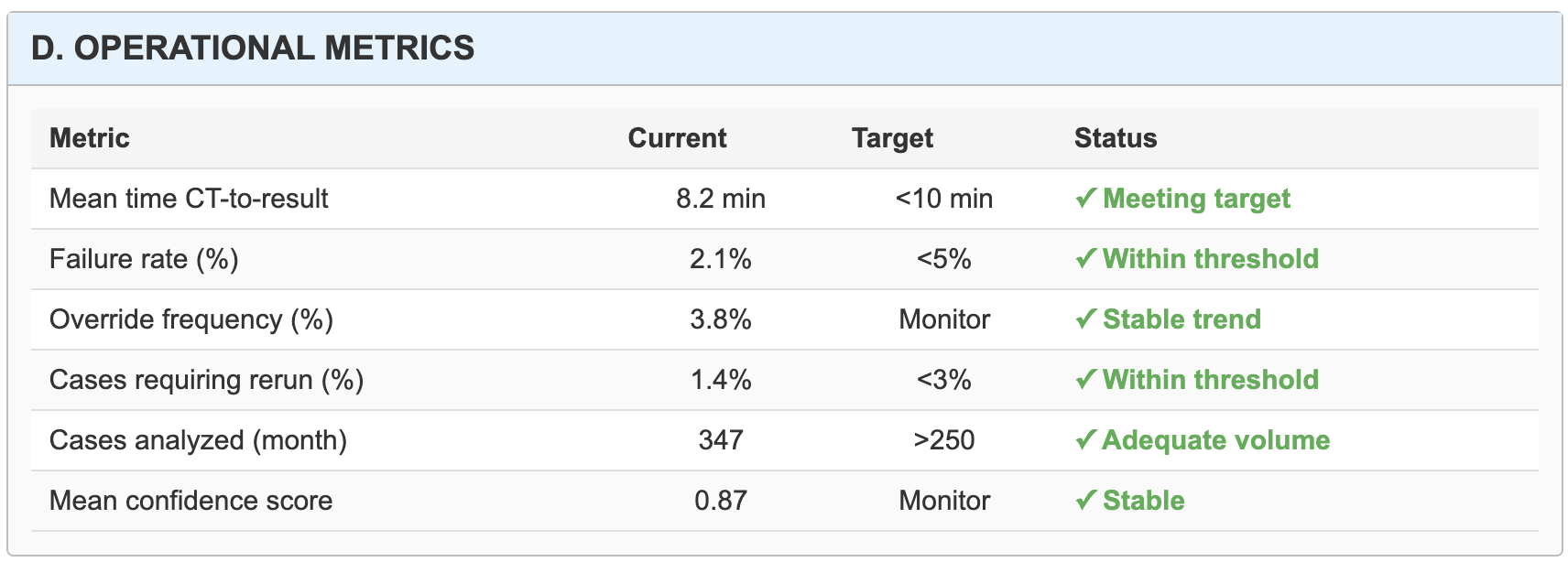 |
| 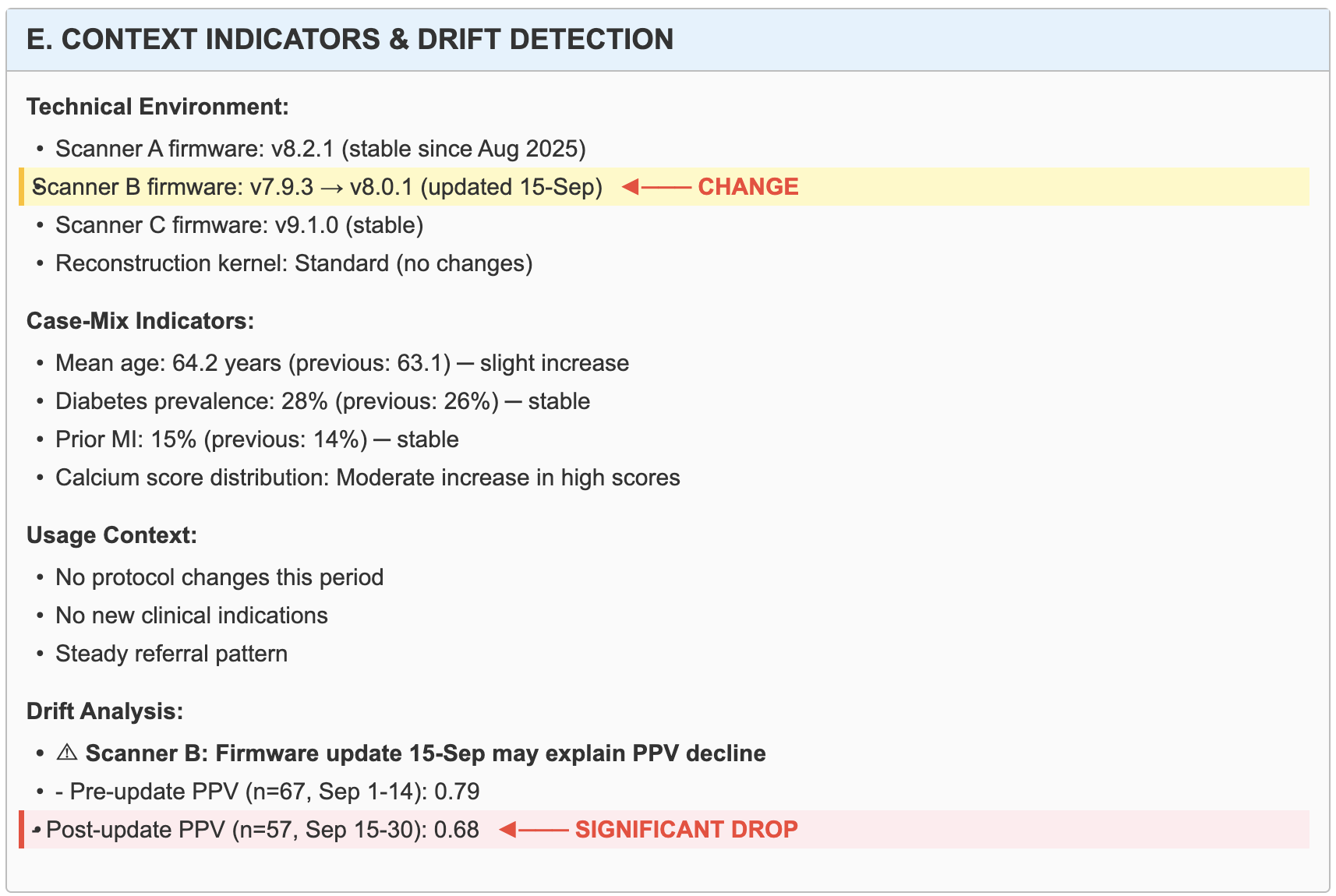 |
| 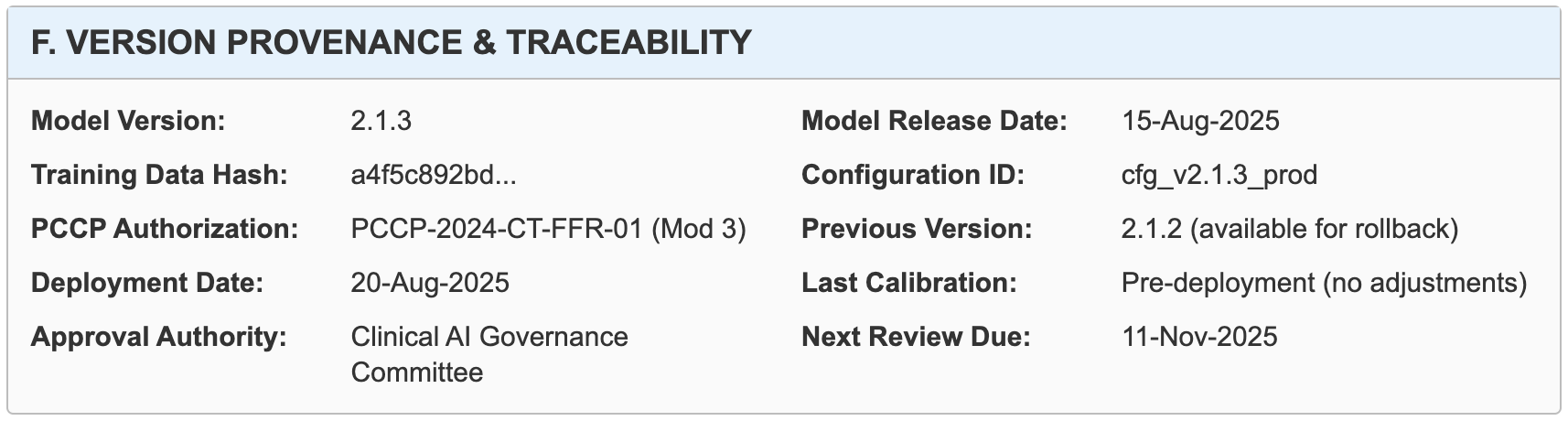 |
| 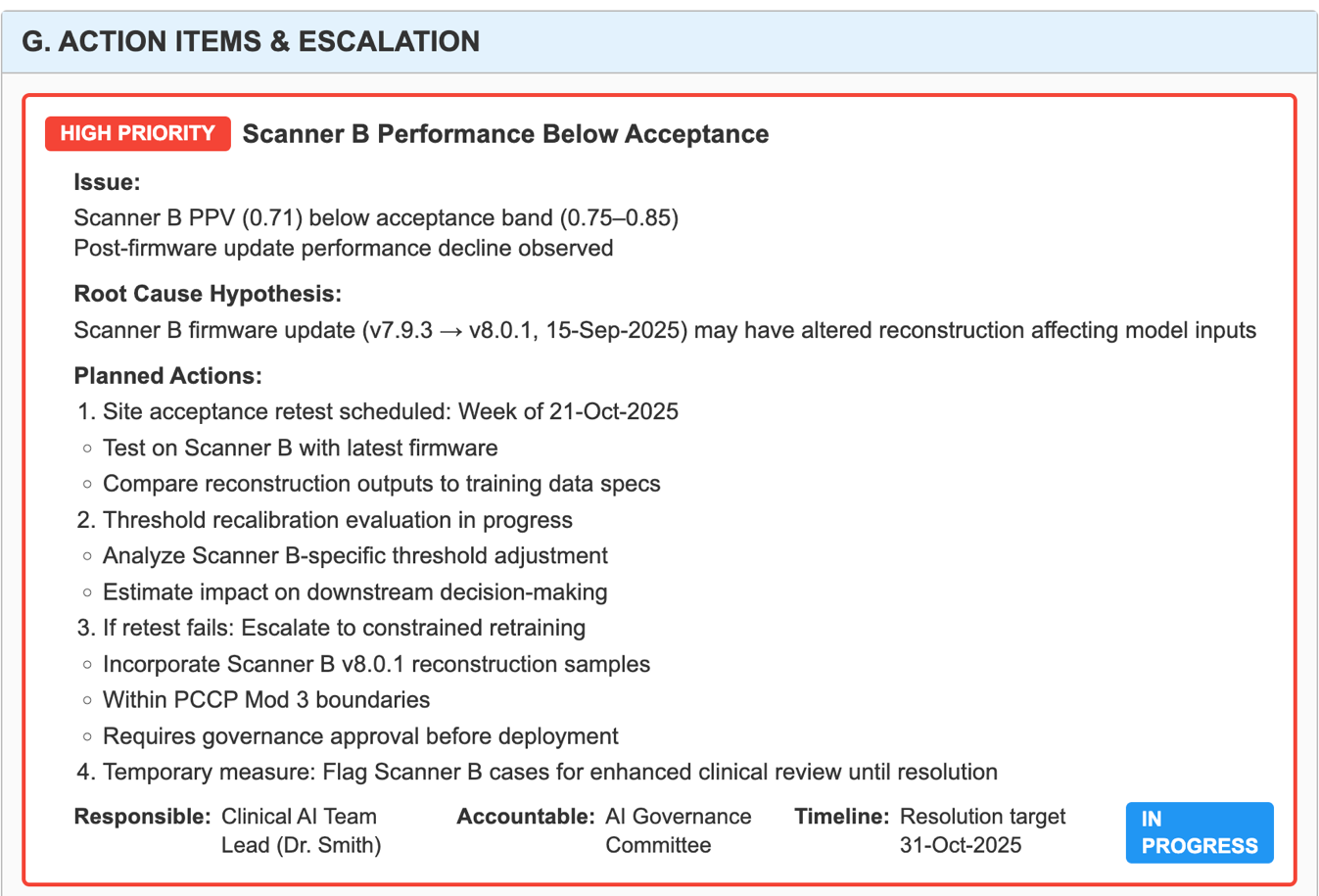 |
| 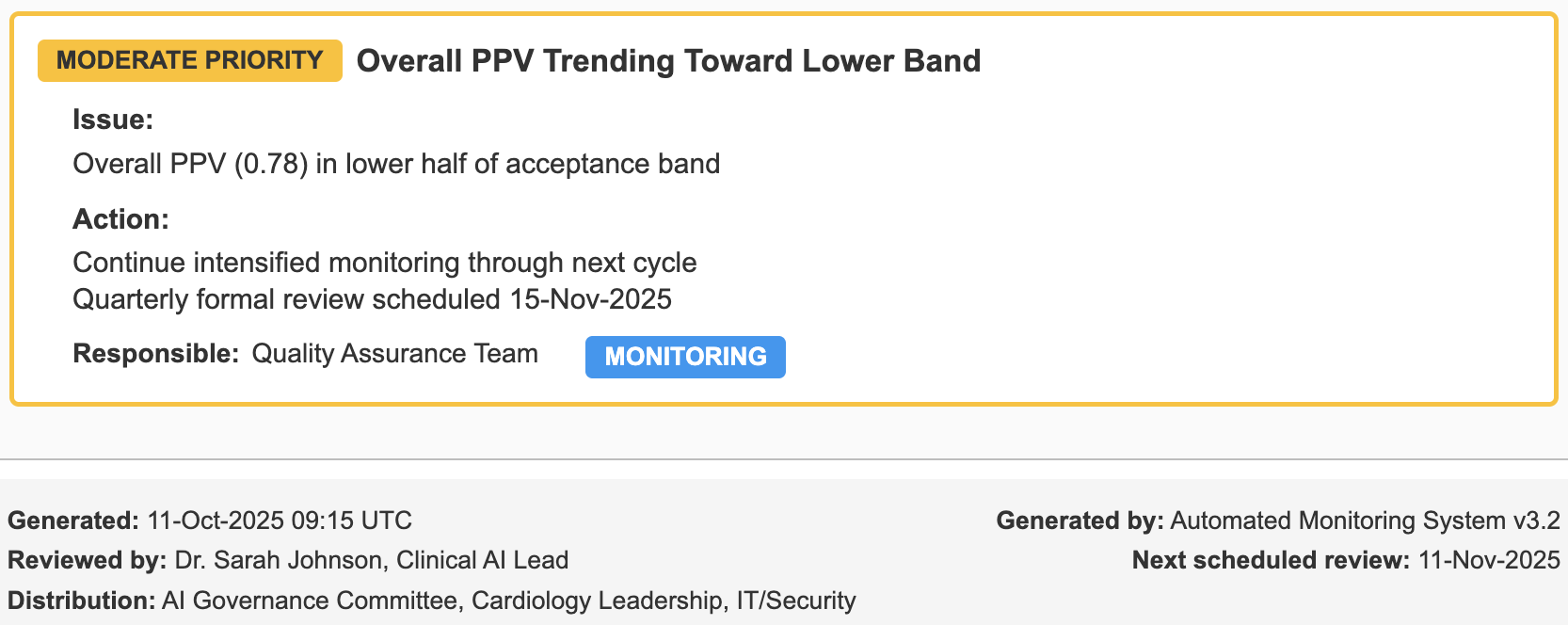 |

**Supplementary Figure S1**. Unified monitoring dashboard framework for AI-enabled cardiovascular devices (illustrative example: CT-FFR). The dashboard integrates seven interconnected panels serving both regulatory reporting requirements and internal governance. **Panel A (Performance Metrics)** displays current versus baseline values for key performance indicators with prespecified acceptance bands and status indicators: AUROC (discrimination), calibration slope and intercept, Brier score (overall accuracy), threshold-specific PPV/NPV at operational cutoff (CT-FFR ≤0.80), and net benefit at threshold. Status coding: green checkmark (✓) indicates in range, yellow warning (⚠) indicates monitor closely, red alert (⚠⚠) indicates action required. **Panel B (Calibration Plot)** visualizes observed versus predicted probabilities across prediction range, showing perfect calibration reference line (slope=1.0, intercept=0.0) and current model performance (slope=1.02, intercept=-0.05) with prediction distribution histogram. **Panel C (Subgroup Performance)** stratifies PPV at operational threshold (0.80) by sex, age, ethnicity, and scanner vendor with sample sizes, acceptance bands (wider for smaller samples), and status indicators. Example shows Scanner B performance below acceptance threshold (0.71 vs. 0.75-0.85 band) triggering action pathway. **Panel D (Operational Metrics)** tracks mean time CT-to-result, failure rate, override frequency, cases requiring rerun, monthly case volume, and mean confidence score against operational targets. **Panel E (Context Indicators & Drift Detection)** monitors technical environment (scanner firmware versions with change flagging), case-mix indicators (demographics, comorbidities, calcium scores), usage context (protocols, indications, referral patterns), and drift analysis identifying potential root causes. Example identifies Scanner B firmware update (v7.9.3 → v8.0.1 on 15-Sep) temporally associated with PPV decline from 0.79 pre-update to 0.68 post-update. **Panel F (Version Provenance & Traceability)** documents model version, release date, training data hash, configuration ID, PCCP authorization, previous version availability for rollback, deployment date, calibration status, approval authority, and next review date. **Panel G (Action Items & Escalation)** prioritizes issues with root cause hypotheses, planned corrective actions (site acceptance retest, threshold recalibration evaluation, potential constrained retraining within PCCP boundaries, temporary enhanced clinical review), RACI assignments (Responsible, Accountable, Consulted, Informed), timelines, and status tracking. Example shows high-priority escalation for Scanner B with planned site acceptance retest week of 21-Oct-2025, threshold recalibration evaluation in progress, and potential constrained retraining if retest fails. Dashboard generated by automated monitoring system, reviewed by clinical AI lead, distributed to AI governance committee, cardiology leadership, and IT/security. Dashboard serves PMS/PMCF (Post-Market Surveillance/Post-Market Clinical Follow-up) and AI Act logging/monitoring requirements while supporting internal governance. CT-FFR = Computed tomography-derived fractional flow reserve; AUROC = Area Under the Receiver Operating Characteristic Curve; PPV = Positive Predictive Value; NPV = Negative Predictive Value; PCCP = Predetermined Change Control Plan; MI = Myocardial Infarction. Not real patient data.
